# Gene expression of *S100a8/a9* predicts *Staphylococcus aureus*-induced septic arthritis in mice

**DOI:** 10.3389/fmicb.2023.1146694

**Published:** 2023-06-15

**Authors:** Meghshree Deshmukh, Santhilal Subhash, Zhicheng Hu, Majd Mohammad, Anders Jarneborn, Rille Pullerits, Tao Jin, Pradeep Kumar Kopparapu

**Affiliations:** ^1^Department of Rheumatology and Inflammation Research, Institute of Medicine, Sahlgrenska Academy, University of Gothenburg, Gothenburg, Sweden; ^2^Cold Spring Harbor Laboratory, Cold Spring Harbor, NY, United States; ^3^Center for Clinical Laboratories, The Affiliated Hospital of Guizhou Medical University, Guiyang, China; ^4^Department of Rheumatology, Sahlgrenska University Hospital, Gothenburg, Sweden; ^5^Department of Clinical Immunology and Transfusion Medicine, Sahlgrenska University Hospital, Gothenburg, Sweden

**Keywords:** septic arthritis, *S100a8/a9*, biomarker, *Staphylococcus aureus*, RNA sequencing, mice

## Abstract

Septic arthritis is the most aggressive joint disease associated with high morbidity and mortality. The interplay of the host immune system with the invading pathogens impacts the pathophysiology of septic arthritis. Early antibiotic treatment is crucial for a better prognosis to save the patients from severe bone damage and later joint dysfunction. To date, there are no specific predictive biomarkers for septic arthritis. Transcriptome sequencing analysis identified *S100a8/a9* genes to be highly expressed in septic arthritis compared to non-septic arthritis at the early course of infection in an *Staphylococcus aureus* septic arthritis mouse model. Importantly, downregulation of *S100a8/a9* mRNA expression at the early course of infection was noticed in mice infected with the *S. aureus* Sortase A/B mutant strain totally lacking arthritogenic capacity compared with the mice infected with parental *S. aureus* arthritogenic strain. The mice infected intra-articularly with the *S. aureus* arthritogenic strain significantly increased *S100a8/a9* protein expression levels in joints over time. Intriguingly, the synthetic bacterial lipopeptide Pam2CSK4 was more potent than Pam3CSK4 in inducing *S100a8/a9* release upon intra-articular injection of these lipopeptides into the mouse knee joints. Such an effect was dependent on the presence of monocytes/macrophages. In conclusion, *S100a8/a9* gene expression may serve as a potential biomarker to predict septic arthritis, enabling the development of more effective treatment strategies.

## 1. Introduction

Septic arthritis is the most serious joint disease primarily caused by the gram-positive bacteria *Staphylococcus aureus* (*S. aureus*) (Goldenberg, 1998; Dubost et al., 2002; Ali et al., 2015; Fatima et al., 2017; Fei et al., 2022; Momodu and Savaliya, 2022). In western Europe, the annual incidence of septic arthritis is 4–10 per 100,000 patients (Kaandorp et al., 1997; Weston et al., 1999; Geirsson et al., 2008; Gunnlaugsdóttir et al., 2022). The mortality rate of septic arthritis is 5–15%, and 25–50% of patients experience a permanent loss of joint function (Goldenberg, 1998; Tarkowski, 2006). The prevalence of septic arthritis appears to be increasing, and this rise is associated with an increase in orthopedic-related infections (Gunnlaugsdóttir et al., 2022). In patients with underlying joint diseases such as rheumatoid arthritis, the prevalence of septic arthritis is 10 times higher (Tarkowski, 2006). In the past few decades, no significant improvement in treatments to prevent joint dysfunction in septic arthritis has occurred (Shirtliff and Mader, 2002). Most cases of septic arthritis are caused by the hematogenous spreading of bacteria into the joint cavities (Carpenter et al., 2011). This is followed by the rapid activation of the immune system and recruitment of immune cells to the joints, causing bone and cartilage damage (Mohammad et al., 2019, 2020).

Inflammation is a basic defense mechanism in the human body against infection. *S100a8* (also known as Myeloid related protein, MRP8) and *S100a9* (MRP14) belong to the S100 family and usually exist as heterodimeric complexes (*S100a8/a9*) due to their instability in the form of homodimer (Manitz et al., 2003). *S100a8/a9*, also known as calprotectin, forms heterotetramers when exposed to calcium ions (Ca^2+^) (Strupat et al., 2000; Donato, 2007). *S100a8/a9* is expressed in neutrophils, monocytes, macrophages, and dendritic cells, which modifies the inflammatory response by promoting leukocyte recruitment and increased cytokine production (Odink et al., 1987; Averill et al., 2011; Schneider et al., 2016; Wang et al., 2018; Blom et al., 2020). Approximately 45% of the cytoplasmic proteins in neutrophils are *S100a8* and *S100a9* proteins. As a Ca^2+^ sensor, *S100a8/a9* is involved in the cytoskeleton’s reorganization and arachidonic acid metabolism (Rammes et al., 1997; Kerkhoff et al., 1999; Vogl et al., 2004). *S100a8/a9* binds to receptors including Toll-like receptor 4 (TLR4) and the receptor for advanced glycation end products (RAGE), which activates NF-κB signaling and consequently causes an inflammatory response (Donato, 2007; Vogl et al., 2007; Ma et al., 2017). During infection, excessive *S100a8/a9* expression intensifies the inflammatory response and speeds up the release of cytokines by neutrophils and macrophages, which creates a vicious cycle and worsens the disorder (Guo et al., 2021; Mellett and Khader, 2022). *S100a8/a9* is necessary to induce autoreactive CD8+ T lymphocytes in autoimmune diseases, which causes inflammation mediated by TLR4 signaling and results in elevated interleukin-17 (IL-17) (Vogl et al., 2007; Loser et al., 2010). Aberrant alterations of *S100a8/a9* are well noted for many diseases including cancer (Duan et al., 2013; Kwon et al., 2013; Koh et al., 2019), cardiovascular (Boyd et al., 2008; Sreejit et al., 2019), skin diseases (Schonthaler et al., 2013), autoimmune disorders (Ometto et al., 2017; Vogl et al., 2018; Defrêne et al., 2021), infections (Scott et al., 2020), and many more (van Lent et al., 2010; Wang et al., 2014).

Several lines of evidence suggest the crucial role of *S100a8/a9* in arthritis diseases. *S100a8* was shown to upregulate Fcγ receptors through TLR4 activation in synovium during chronic experimental arthritis (van Lent et al., 2010). The interaction of monocytes with inflamed endothelium results in the release of *S100a8/a9* in juvenile rheumatoid arthritis (Frosch et al., 2000). In rheumatoid arthritis, *S100a8/a9* produced by activated macrophages may increase cytokine production via activating the NF-κB and p38 MAPK pathways (Sunahori et al., 2006). *S100a8/a9*, as a biomarker, has been intensively studied for many diseases including inflammatory bowel disease (Pruenster et al., 2016), myocarditis (Müller et al., 2020), rheumatoid arthritis (Frosch et al., 2000; Inciarte-Mundo et al., 2022), cystic fibrosis (Cohen and Larson, 2005), and infections (Fang et al., 2021). Delayed diagnosis of septic arthritis is strongly linked with severe joint damage and permanent joint dysfunction among patients with this debilitating joint disease. A biomarker predicting septic arthritis at the time of systemic infection may improve the diagnostics, reduce the treatment delay, and finally significantly reduce permanent joint dysfunction. Here, from our next-generation studies on animal models of *S. aureus*-induced septic arthritis, we found that *S100a8/a9* gene expression is one of the top candidates that could be used as a biomarker to predict septic arthritis. Furthermore, we validated our RNAseq data in the septic arthritis mouse model using both the arthritogenic *S. aureus* (Newman) strain and the non-arthritogenic (*SrtA/B* mutant (Δ*srtA/B*)) strain. Overall, our data suggest that *S100a8/a9* might be used as a biomarker to predict septic arthritis before the debut of clinical arthritis symptoms in a systemic *S. aureus* infection.

## 2. Materials and methods

### 2.1. Mice

NMRI and C57BL/6 mice, aged 6–9 weeks, were purchased from Envigo (Venray, Netherlands). All mice were housed at the animal facility at the University of Gothenburg. Mice were kept under standard temperature and light conditions and were fed laboratory chow and water *ad libitum*. As prior studies did not reveal a noteworthy impact of sex difference on the outcomes of *S. aureus* hematogenous septic arthritis in mouse models (Hu et al., 2023), we opted to exclusively employ female mice in current study. The Ethics Committee of Animal Research of Gothenburg approved the study, and the animal experimentation guidelines of the Swedish Board of Agriculture were strictly followed.

### 2.2. Bacterial strains

#### 2.2.1. *Staphylococcus aureus* strains

Newman wild type (WT) (Duthie and Lorenz, 1952), bioluminescent Newman strain (AH5016) (Miller et al., 2019) and Newman Δ*srtA/B* that lacks Sortase A/B expression (Mazmanian et al., 2002). All strains were cultivated separately for 24 h on horse blood agar plates or trypticase soy agar (TSA) plates with erythromycin (2.5 μg/mL), respectively, and then preserved as previously mentioned (Mohammad et al., 2020). Before each experiment, the bacterial solutions were thawed, washed, and adjusted to the desired concentration for the respective experiments.

### 2.3. *In vivo* mice experiments

**Figure 1:**
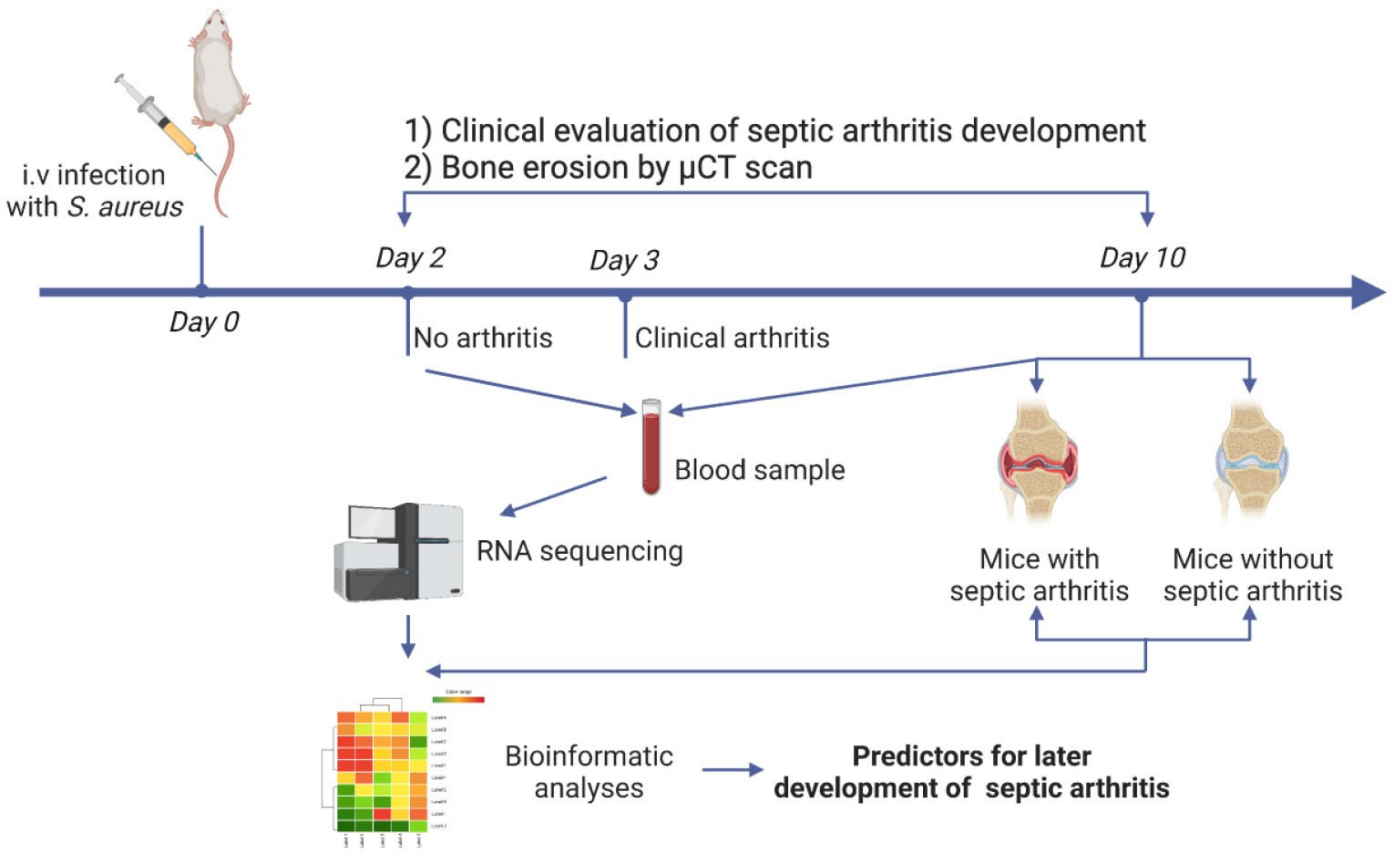
Schematic presentation of experimental design. NMRI mice (*n* = 20) were intravenously infected with the *Staphylococcus aureus* Newman wild-type (WT) strain. Blood was collected on day 2 and day 10 while the joints were collected on day 10 post-infection. Development of clinical arthritis started from day 3 and followed until day 10. RNA from blood samples taken on day 2 and day 10 were sequenced. Joints were scanned with micro-computed tomography (μCT) for evaluating bone erosions.

The experimental setting for identification of the predictors for septic arthritis is demonstrated in Figure 1. For the modeling experiment, mice (*n* = 20) were intravenously (i.v.) infected with 200 μL of the Newman WT strain (2 × 10^7^ CFU/mouse). In our animal model, the clinical signs of septic arthritis usually develop on days 3–5 after i.v injection of *S. aureus*. Therefore, we chose day 2 to collect the blood samples to study the predictive biomarkers for septic arthritis. On day 2 post-infection the blood was drawn from the tail vein of the mice and RNA was extracted for the later transcriptome analysis. The weight loss and clinical arthritis were followed twice a day until day 10 post-infection. The joints were collected for later microcomputed tomography (micro-CT or μCT) analyses. The mice with either clinical arthritis or bone erosions on micro-CT were classified as septic arthritis mice. The mice who never had any clinical signs of septic arthritis or bone erosions on micro-CT were deemed as non-septic arthritis mice.

For the validation of the i.v. study, 200 μL of Newman WT suspension (6 × 10^5^ CFU/mouse; *n* = 15) or Newman Δ*srtA/B* suspension (6 × 10^5^ CFU/mouse; *n* = 5) was inoculated i.v. into the tail vein of each respective set of NMRI mice. The animals were monitored by three observers (M.D., P.K.K., and Z.H.) for up to 10 days after infection. Mice were monitored and assessed to estimate the severity of arthritis, and a clinical scoring system ranging from 0 to 3 was used as previously described (Fatima et al., 2017). Blood samples were collected from the surviving mice on days 2, 7, and 10 after infection. At day 10 post-infection, the blood and kidneys were collected followed by paws for microcomputed tomography (micro-CT). Kidney abscesses were assessed by two investigators (M.D., and M.M.) in a blinded manner. The kidneys were then homogenized and plated on horse blood agar plates to quantify the CFUs.

200 μL of Newman WT suspension (8 × 10^5^ CFU/mouse) was inoculated i.v. into the tail vein of C57BL/6 mice (*n* = 10). The severity of arthritis and weight loss were followed. Blood samples were collected on days 2 and 10 after infection.

In the local knee model, to induce septic arthritis in NMRI mice (*n* = 9), 20 μL of *S. aureus* Newman WT suspension 2 × 10^4^ CFU/knee was injected intra-articularly (i.a.) in the knee joints. The animals were monitored by three observers (M.D., P.K.K., and Z.H.) for up to 9 days after infection. In another experiment, the synthetic lipopeptides Pam2CSK4 (EMC, Tübingen, Germany; 4 μg/knee) and Pam3CSK4 (EMC, Tübingen, Germany; 4 μg/knee), mimicking the lipid portion of bacterial lipoproteins, were administered locally in the NMRI mouse knee joints (*n* = 16); The mice were divided into 2 groups (Pam2CSK4 and Pam3CSK4) with two time points (day 1 and day 3). Four mice were sacrificed on day 1 and four mice were sacrificed on day 3 for sample collection. PBS injected i.a. mice served as controls (*n* = 2, 4 joints). The animals were monitored by two observers (Z.H. and T.J.) for up to 3 days after injection. The diameter of the knee joints of mice were measured regularly with a caliper to determine the severity of the induced arthritis.

In another experiment, as previously mentioned (Mohammad et al., 2019; Kopparapu et al., 2021) to deplete both synovial macrophages at the joints and systemic monocytes, NMRI mice (*n* = 3, 6 joints) were i.a. injected with 20 μL of clodronate liposomes (Liposoma BV, Amsterdam, Netherlands) (Van Rooijen and Sanders, 1994) into the knee joints as well as i.v. injected with 200 μL of clodronate liposomes, while PBS control liposomes served as controls (*n* = 3, 6 joints) (Liposoma BV, Amsterdam Netherlands). After 24 h of depletion, mice were i.a. injected with Pam2CSK4 (4 μg/knee) in the knee joints and monitored by two observers (Z.H. and T.J.) for up to 3 days after injection. Mice were weighed and the severity of the clinical arthritis was judged by measuring the difference between the diameters of the knee joints with a caliper daily (Z.H. and T.J.).

In the experiment with antibiotic treatment, NMRI mice (*n* = 10) were infected i.v. with *S. aureus* Newman bioluminescent strain (AH5016) suspension with a dose of 2.8 × 10^6^ CFU/mouse. After day 3 post-infection, the mice (*n* = 5) were treated subcutaneously with cloxacillin (10 mg/mouse) in 0.2 mL of PBS twice per day until day 9 when mice were sacrificed. Same volume of PBS was given to the control mice. The cloxacillin dose was well established to eradicate the *S. aureus* in our septic arthritis mouse model (Fei et al., 2011). At day 9 post-infection, the mice were examined by *In Vivo* Imaging System (IVIS) to compare the live bacteria load in different organs, such as joints and kidneys, between the cloxacillin treatment group and the PBS control group. The blood cells and plasma were collected for further processing, while joints were collected for micro-CT scan.

### 2.4. RNA extractions and RNA sequencing

RNA extractions were done on blood using miRNeasy Mini Kit (Qiagen, Hilden, Germany) according to the manufacturer’s instructions. The quantity and quality of isolated RNA were determined using the NanoDrop 2000 Spectrophotometer (Thermo Fischer Scientific, Waltham, USA), Qubit^®^ RNA HS Assay Kit (Invitrogen, Waltham, USA), and 2200 TapeStation Automated Electrophoresis System (Agilent Technologies, Santa Clara, USA). The samples had RNA integrity numbers between 2.2 and 8.8.

Ribosomal RNA was removed, and sequencing libraries were prepared using the TruSeq Stranded Total RNA Sample Preparation Kit with Ribo-Zero Gold (Illumina, San Diego, USA), following the manufacturer’s instructions. Construction of libraries was prepared using the TruSeq Stranded Total RNA Sample Preparation Guide 15,031,048 Rev. E (Illumina, San Diego, USA), using 0.5–1 μg of total RNA input. The Novaseq 6000 platform was used and 100 bp paired-end reads were generated by Clinical Genomics at the University of Gothenburg (Gothenburg, Sweden).

### 2.5. Transcriptome analysis of septic arthritis

The paired-end reads with strand-specific library (fr-first strand) preparation of samples from this study were aligned using HISAT2 (v2.2.1) (Kim et al., 2015) against reference ensemble mouse genome GRCm39 (Cunningham et al., 2022). Obtained alignment files are indexed and sorted using SAMtools (v1.5) (Danecek et al., 2021). These alignment files were further used for gene quantification using GENCODE (Frankish et al., 2021) gene annotation corresponds to the GRCm39 genome. featureCounts from subread (v2.0.0) (Liao et al., 2014) package is used for assigning high-quality uniquely mapped reads to the gene features with paired-end and strand-specific parameters (−p-s 2-B --minOverlap 10-Q 30 --ignoreDup). Differentially expressed genes between different biological conditions were obtained using the DESeq2 Bioconductor package (R v4.1.1) (Love et al., 2014). The significant differential genes were filtered based on an adjusted value of *p* less than 0.01 and the absolute log-fold difference between two comparison groups of 1. Obtained differentially expressed genes were subjected to functional enrichment analysis against KEGG and gene ontology databases using GeneSCF (v1.1-p2) (Subhash and Kanduri, 2016). Significant pathways are filtered using a value of *p* less than 0.05.

### 2.6. cDNA synthesis and quantitative RT-PCR

The total cDNA synthesis was done by using a Superscript III First-Strand synthesis supermix kit (Invitrogen, Waltham, USA). The expression levels of *S100a8* and *S100a9* were analyzed by quantitative RT-PCR using Power SYBR Green gene expression assays (Applied Biosystems, Warrington, UK), wherein, mouse *β-actin* (*Actb*) was used as an internal control. The predesigned KiCqStart primers (KiCqStart SYBR Green, Merck, Darmstadt, Germany) were used. Differences in expression were calculated using the ΔCt method. The primer details are as follows: *S100a8* (FP 5’ ATACAAGGAAATCACCATGC 3′) (RP 5’ ATATTCTGCACAAACTGAGG 3′); *S100a9* (FP 5’ CTTTAGCCTTGAGCAAGAAG 3′) (RP 5’ TCCTTCCTAGAGTATTGATGG 3′); *β*-*actin* (FP 5’ GATGTATGAAGGCTTTGGTC 3′); (RP 5’ TGTGCACTTTTATTGGTCTC 3′).

### 2.7. Enzyme-linked immunosorbent assay

Knee joint from the i.a. experiments was homogenized using TissueLyser II (Qiagen, Hilden, Germany) while blood plasma was collected from C57BL/6 or NMRI mice infected intravenously with Newman WT and Newman Δ*srtA/B*. The levels of *S100a8/a9* in plasma and knee homogenates were analyzed using an *S100a8/a9* Heterodimer DuoSet ELISA kit (R&D Systems, Abingdon, UK) as per the manufacturer’s instructions.

### 2.8. Western blot

Western blotting was conducted with the plasma and cell lysates of mouse blood samples according to the standard manufacturer’s protocol. All of the samples were resolved on Tricine 10–20% gel (Invitrogen, Carlsbad, USA) along with a molecular mass marker (Chameleon Duo pre-stained protein ladder 8 kDa – 250 kDa, LI-COR Biosciences –, Lincoln, USA) and transferred to nitrocellulose membranes (TransBlot Turbo Mini-size nitrocellulose, Bio-Rad, Hercules, USA) using semi-dry method (TransBlot Turbo Transfer System, Bio-Rad, Hercules, USA). After blocking with 3% BSA (Sigma-Aldrich, St. Louis, USA) membrane was treated with anti *S100a9* polyclonal antibody (PA1-46489, Invitrogen, Carlsbad, USA) as primary antibody, washed with 1X TBST (Thermo Fischer Scientific, Waltham, USA), and incubated with secondary antibody (IRDye 680RD Goat anti-Rabbit IgG, LI-COR Biosciences, Lincoln, USA). After washing, the blot was visualized with Odyssey XF Imaging System (LI-COR Biosciences, Lincoln, USA).

### 2.9. Microcomputed tomography (μCT)

Joints were fixed in 4% formaldehyde for 3 days and then transferred to PBS for 24 h. All four paws were scanned by SkyScan 1,176 μCT (Bruker, Antwerp, Belgium). The scanning was conducted at 55 kV/ 455 μA, with a 0.2-mm aluminum filter. The exposure time was 47 ms. The X-ray projections were obtained at 0.7° intervals with a scanning angular rotation of 180°. The NRecon software (version 1.6.9.8; Bruker) was used to reconstruct three-dimensional images and evaluated by using CT Analyzer (version 2.7.0; Bruker). Each joint was evaluated by two researchers (M.D. and T.J.) using a scoring system from 0 to 3 (0: healthy joint; 1: mild bone destruction; 2: moderate bone destruction; and 3: marked bone destruction) as previously described (Fatima et al., 2017).

### 2.10. *In vivo* imaging system

The mice were anesthetized with an intraperitoneal injection of 200 μL of ketamine/xylazine mixture and examined by Newton 7.0 FT500 *In Vivo* Bioluminescence Imaging System (Vilber Lourmat, Marne-la-Valée, France). Bioluminescent signals from dorsal side of the mice were captured for 5 min at 37°C with sensitivity of ultimate XL (8) using Evolution-Capt software (Vilber Lourmat). Image analysis was carried out using Kuant software (Vilber Lourmat Marne-la-Valée, France).

### 2.11. Statistical analysis

All results are reported as the mean ± standard error of the mean (SEM) if not indicated otherwise and a *p*-value <0.05 was considered statistically significant. All nonparametric data sets were analyzed using the Mann–Whitney *U* test and Fisher’s exact test. Statistical analyses were performed using GraphPad Prism version 9 (GraphPad Software, La Jolla, USA).

## 3. Results

### 3.1. *S100a8/a9* gene expression involved in septic arthritis pathogenesis

**Figure 2:**
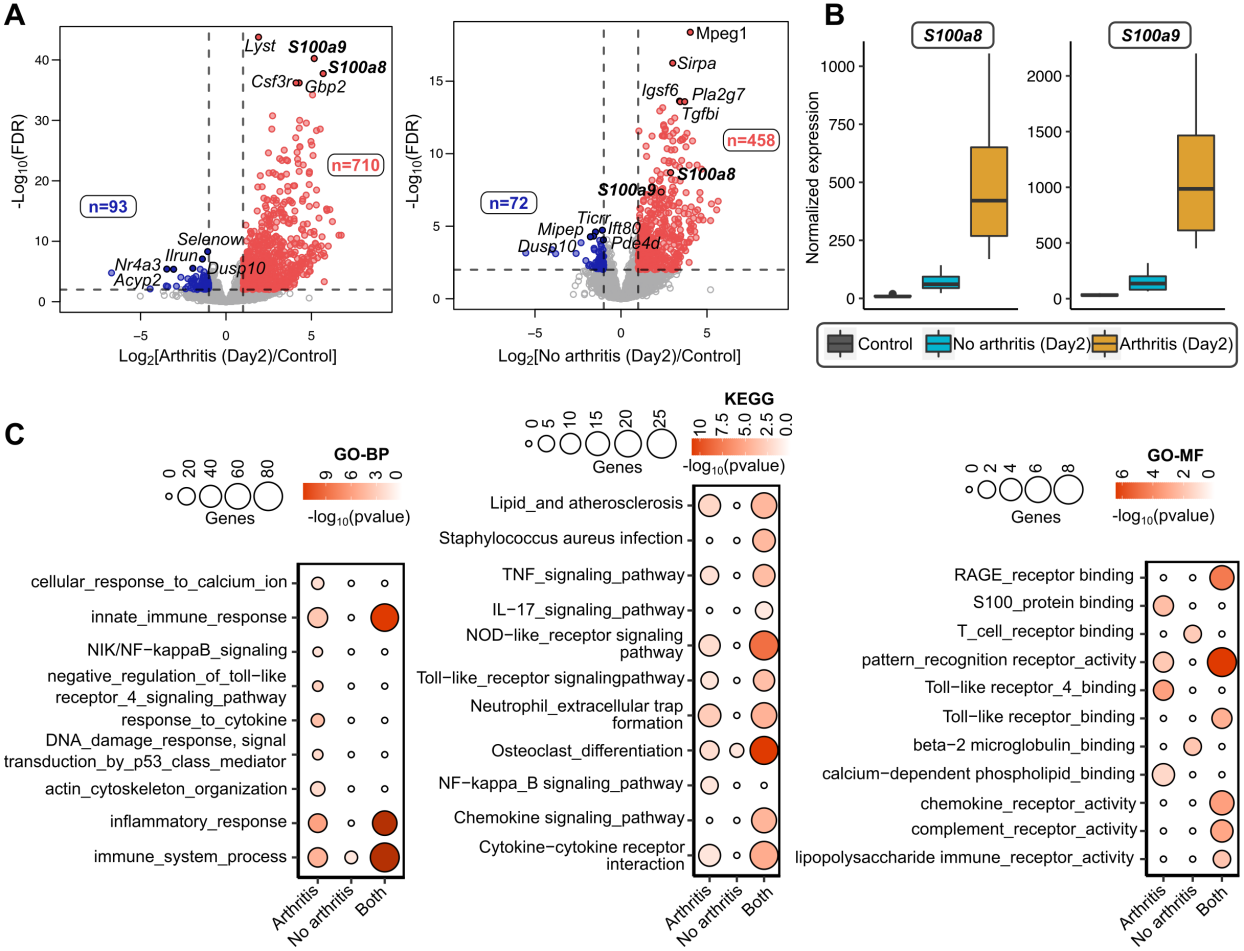
Transcriptome analysis of septic arthritis mice on day 2 after *S. aureus* infection. **(A)** Volcano plots showing differentially expressed genes between day 0 mice (healthy controls) and day 2 mice developed arthritis (left) and without arthritis (right) after *S. aureus* infection. The horizontal dotted line denoted a false discovery rate (FDR) cut-off of 0.05 and the vertical dotted lines denote a log-fold difference cut-off of ±1. The red and blue color indicates up-regulated and down-regulated genes, respectively, in arthritis (left) or non-arthritic mice (right) compared to healthy controls (day 0). **(B)** Boxplots comparing normalized expression patterns between day 0 and day 2 (mice with and without arthritis) after infection. **(C)** Gene Ontology (GO-BP: Biological Process; GO-MF: Molecular Function) and KEGG pathways enriched with arthritis-specific genes and non-arthritic specific genes after infection; and the terms enriched commonly between arthritic and non-arthritic mice. The size of the inner circles represents many differentially expressed genes involved in the enriched terms and the color gradient denotes the statistical significance of individual terms.

To identify the potential gene markers predicting *S. aureus* septic arthritis, we implemented a methodology as described in schematics (Figure 1). For this purpose, RNA from mice that developed arthritis and mice that did not develop arthritis on the 2nd day of the *S. aureus* infection were sequenced. Additionally, as a control experiment, RNA from the healthy mice on the day of infection (Day 0) was sequenced. Comparisons were made between control mice (Day 0) versus arthritis (Day 2) and non-arthritic mice (Day 2). There were 710 and 93 genes up-and down-regulated, respectively, in the mice that developed arthritis; 458 and 72 genes were up-and down-regulated, respectively, in the non-arthritic mice (Figure 2A; Supplementary File S1). Importantly, *S100a8* and *S100a9* were the top genes that distinguished the arthritis mice and non-arthritis mice on the day after infection. The expression levels of *S100a8* and *S100a9* were significantly elevated in arthritic mice compared to non-arthritic mice (Figure 2B). There were significant molecular pathways enriched genes higher in the arthritis mice compared with non-arthritis mice (Figure 2C). Biological process analysis revealed an enrichment of genes specific to septic arthritis that are involved in calcium ion cellular response, NF-κb signaling, TLR4 signaling pathway, cytokine response, actin cytoskeleton organization, etc., whereas the KEGG process revealed arthritis-specific enriched genes involved in NF-κb signaling. Gene enrichment of S100 protein binding, TLR4 binding, calcium-dependent phospholipid binding molecular functional pathways involved specifically to septic arthritis (Figure 2C; Supplementary File S2–S4).

### 3.2. *S100a8*/*a9* Gene expression acts as an early predictor for septic arthritis in mice

**Figure 3:**
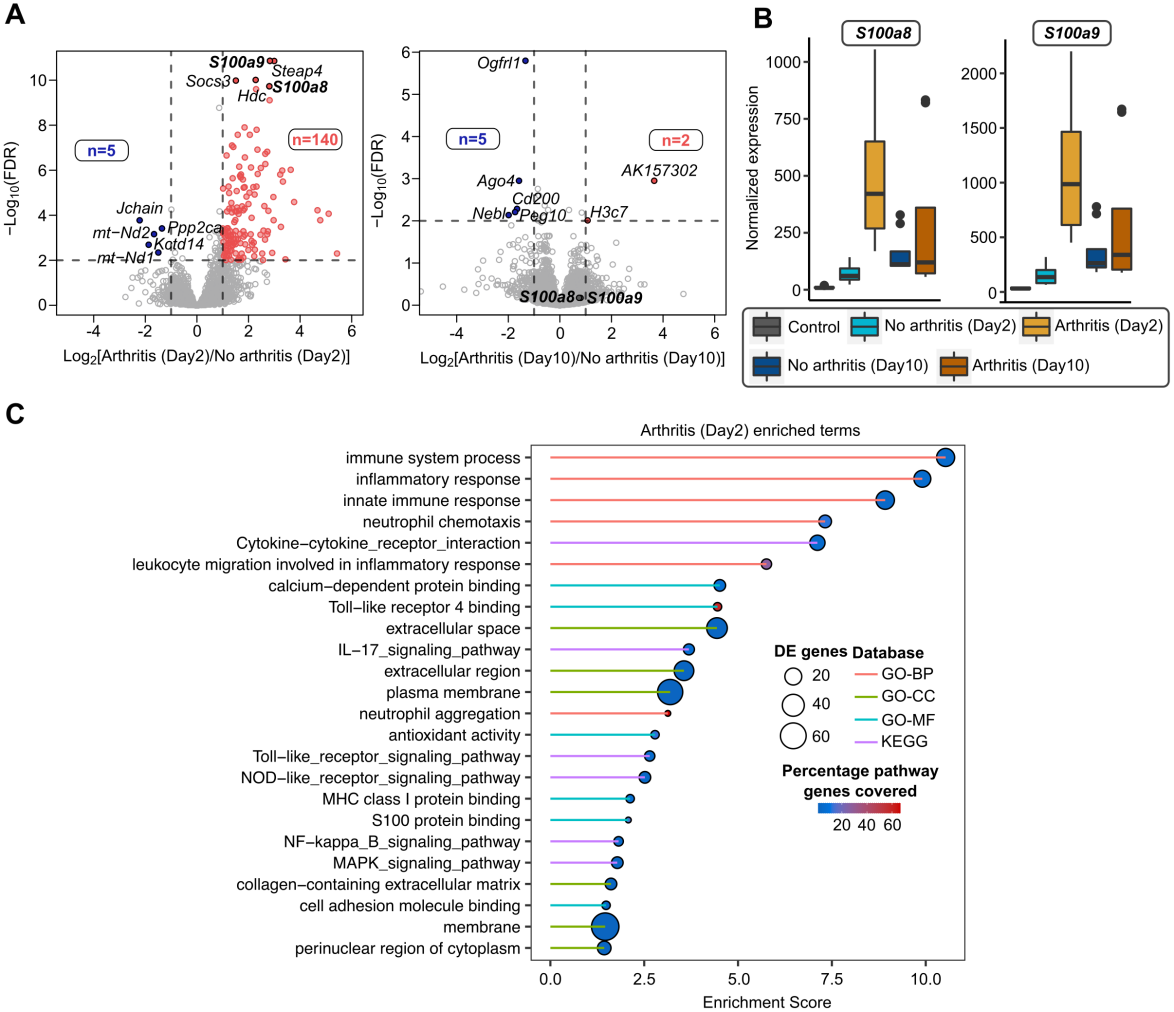
Transcriptome analysis of septic arthritis mice on day 2 compared with day 10 after *S. aureus* infection. **(A)** Volcano plots showing differentially expressed genes between arthritic and non-arthritis mice infected with *S. aureus* infection after day 2 mice (left) and day 10 mice (right). The horizontal dotted line denoted a false discovery rate (FDR) cut-off of 0.05 and vertical dotted lines denote a log-fold difference cut-off of ±1. The red and blue color indicates up-regulated and down-regulated genes, respectively, in day 2 arthritic (left) and day 10 arthritic mice (right) compared to non-arthritic mice from respective days. **(B)** Boxplots comparing normalized expression patterns between day 0, day 2 (mice with and without arthritis), and day 10 (mice with and without arthritis) after infection. **(C)** Gene Ontology (GO-BP: Biological Process; GO-CC: Cellular Component; GO-MF: Molecular Function) and KEGG pathways enriched with deregulated genes from day 2 arthritic mice after infection. The size of the circles represents many differentially expressed genes involved in the enriched terms, the color gradient denotes the percentage of genes from the pathways covered by differentially expressed genes, and the line color indicates a different database.

To further study whether *S100a8* and *a9* gene expression at the early stage of bacteremia can predict the later development of septic arthritis, transcriptome sequencing of infected mice at day 2 and day 10 was performed. There were 145 genes deregulated between mice that developed arthritis and the mice without arthritis at day 2 (Figure 3A; Supplementary File S1). Interestingly, *S100a8*/*a9* expression was elevated in mice with arthritis on day 2. At day 10 there was less or minimal difference in transcriptome profiles between mice that developed arthritis and the mice without arthritis (7 deregulated genes) (Figure 3A). *S100a8*/*a9* genes were only deregulated on day 2 but not on day 10 (Figures 3A,B). There were pathways deregulated in septic arthritis mice compared with non-septic arthritis mice (Figure 3C). The top pathways specifically enriched with mice having arthritis were inflammatory and immune response, whereas other major pathways include neutrophil chemotaxis, TLR4, NOD-like, MAPK, NF-κb signaling pathways, etc. (Figure 3C; Supplementary File S5).

### 3.3. Validation of *S100a8/a9* gene expression as a septic arthritis predictor in mice infected with arthritogenic and non-arthritogenic *Staphylococcus aureus* strains

**Figure 4:**
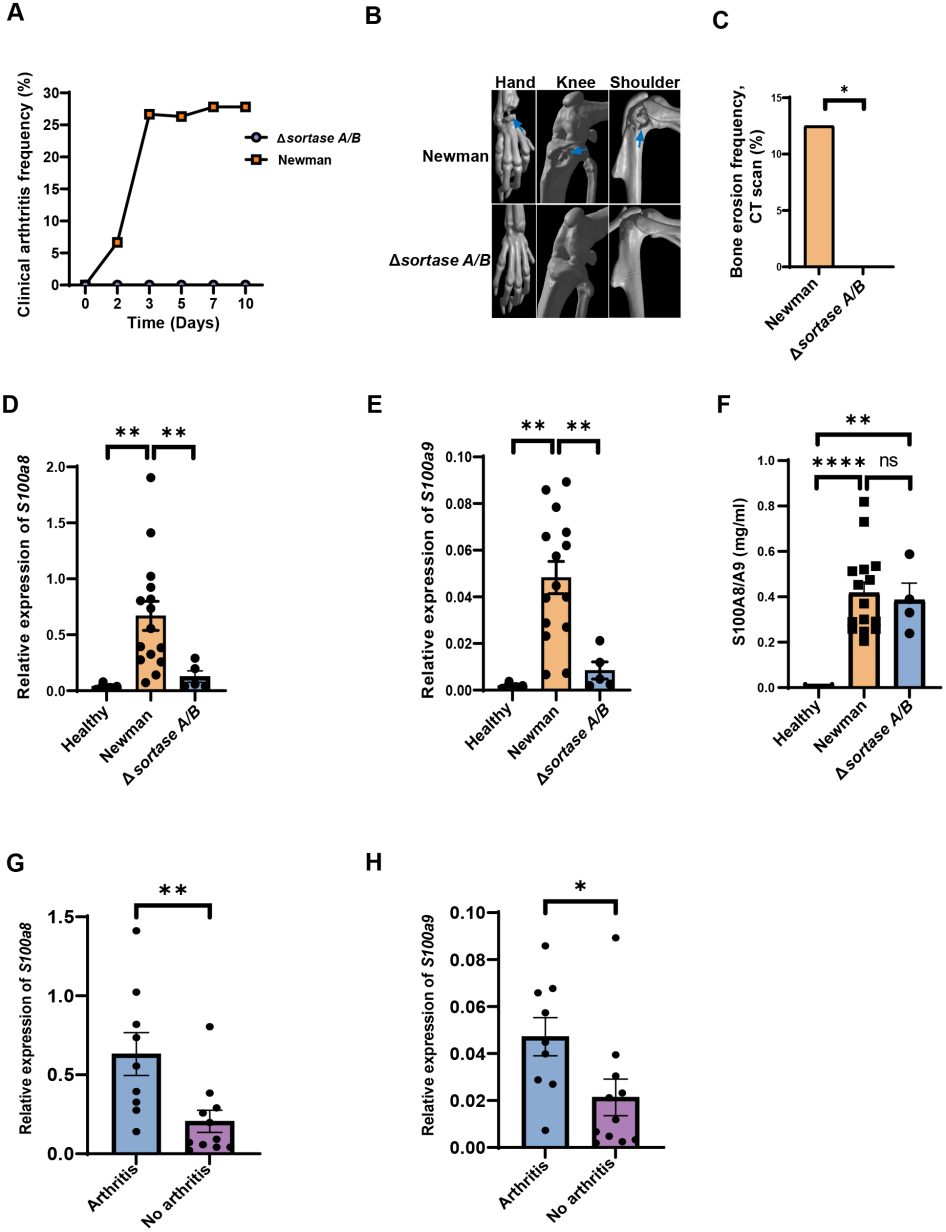
The levels of *S100a8/a9* are dependent on virulence factors. **(A)** Clinical arthritis of the joints in NMRI mice intravenously injected with *Staphylococcus aureus* (*S. aureus*) Newman wild type (WT) strain or Newman Δ*sortase A/B* strain. **(B)** Representative images of micro-computed tomography (μCT) scanning of the mice joints (hand, knee, shoulder; upper panel: Newman; lower panel: Δ*sortase A/B*, arrow: bone erosion) infected with *S. aureus* Newman WT strain or Newman Δ*sortase A/B* strain. **(C)** Bone erosion frequency of joints evaluated using μCT in NMRI mice intravenously injected with *S. aureus* type WT strain or Newman Δ*sortase A/B* strain. Blood mRNA levels of **(D)** *S100a8* **(E)** *S100a9* and **(F)** plasma protein levels of *S100a8/a9* of the mice infected with *S. aureus* Newman WT or Δ*sortase A/B. Blood mRNA levels of* **(G)** *S100a8* and **(H)** *S100a9* between mice with and without septic arthritis on day 2 infected with *S. aureus* Newman WT or Newman Δ*sortase A/B* strain. Statistical analyses were performed using the Fisher’s exact test **(A** & **C)** and Mann– Whitney U test **(D–H)**, where the data were represented in mean ± SEM. *p < 0.05; **p < 0.01; ***p < 0.001; ****p < 0.0001; n.s = not significant.

Sortase enzymes are known to control the *S. aureus* surface proteins anchoring to the bacterial cell wall (Mazmanian et al., 1999). The sortase-deficient *S. aureus* loses its ability to induce septic arthritis (Jonsson et al., 2003). To further validate our findings from RNAseq analysis, we infected mice i.v. with *S. aureus* Newman WT or Newman Δ*srtA/B* and on day 2 after infection, *S100a8* and *a9* gene expression levels in the blood were compared between the groups. Around 30% of mice infected with the *S. aureus* Newman WT strain developed clinical arthritis. In contrast, no mice infected with the *S. aureus* Newman Δ*srtA/B* strain had any sign of septic arthritis (Figure 4A; Supplementary Figure S1A). The mice infected with Newman Δ*srtA/B* strain did not exhibit any bone erosion, whereas the mice with *S. aureus* Newman WT strain group showed joint erosion in 69% of animals (Figures 4B,C; Supplementary Figure S1B). Both groups of mice showed decrease in weight development during the course of infection but there was no significant difference between groups (Supplementary Figure S1C). In total, 13.3% (*n* = 2) of mice died in the Newman WT infected group, while all mice infected with the Newman Δ*srtA/B* mutant strain survived (Supplementary Figure S1D). The kidney abscess score and CFU count were also compared, and no tangible difference was found between those two groups (Supplementary Figures S1E,F).

To further study whether *S100a8* and *a9* gene expression at the early stage of bacteremia can predict the later development of septic arthritis, we compared mRNA levels of *S100a8* and *a9* on day 2 after infection between mice infected with Newman WT strain and mice infected with Δ*srtA/B* strain. Importantly, mRNA levels of *S100a8* and *a9* were significantly higher in mice infected with Newman WT compared with Newman Δ*srtA/B* or with healthy controls (Figures 4D,E). Our data suggest that *S100a8* and *a9* gene expression levels have predictive values for septic arthritis. Additionally, we examined the plasma protein levels of *S100a8/a9* on day 2 after infection in those groups (Figure 4F). *S100a8/a9* was drastically upregulated in Newman WT and Newman Δ*srtA/B* compared to healthy controls. However, no difference was found between WT and mutant strain group (Figure 4F). We conducted a further analysis of plasma protein levels by performing western blot using representative samples from the Newman Δ*srtA/B* strain (3 samples) and the wildtype Newman infected mice on day 2 (3 samples). Our results indicate that all three samples from mice infected with the wildtype strain showed visible protein bands, while none of the samples from the Δ*srtA/B* infected mice had such bands. This suggests that mice infected with the wildtype strain had higher levels of *S100a8/a9* protein than those infected with the Δ*srtA/B* strain (Supplementary Figure S3). Based on these findings, we also conclude that ELISA may not be the optimal method to measure the levels of *S100a8/a9* protein in plasma. In addition, there were significant differences when we analyzed the *S100a8/a9* gene expression levels on day 2 in both septic arthritis mice and mice with infection but without septic arthritis (Figures 4G,H). We conducted further correlation analyses between bone erosion severity on day 10, *S100a8* and *S100a9* gene expression levels, and *S100a8/a9* plasma levels on day 2 (Table 1). Consistent with the data described above, the severity of joint damage shown by μCT significantly and positively correlated with *S100a8* gene expression (*p* < 0.05, *r* = 0.58) and *S100a9* gene expression (*p* < 0.05, *r* = 0.54), but not with *S100a8/a9* plasma levels.

**Table 1:** Spearman’s correlation analysis of bone erosion with mRNA expression of *S100a8*, *S100a9*, and plasma levels of *S100a8/a9*.

	Relative expression of *S100a8*	Relative expression of *S100a9*	Plasma levels of *S100a8/a9*
Correlation coefficient (*r*)	0.582	0.540	−0.129
*p* value	0.0111	0.020	0.616

We also conducted a similar experiment in C57BL/6 mice, where we observed a significant increase in plasma levels of *S100a8/a9* on days 2 and 10 post-infection (Supplementary Figure S2). However, we did not find a significant correlation between plasma levels of *S100a8/a9* on day 2 and the clinical arthritis severity on day 10, suggesting once again that plasma levels of *S100a8/a9* by ELISA method cannot serve as predictors for septic arthritis development. This evidence together with results from RNA sequencing suggest that *S100a8* and *S100a9* gene expression level can predict the outcome of septic arthritis disease pathogenesis.

### 3.4. Elevated *S100a8/a9* levels in joints from mice with local septic arthritis

**Figure 5:**
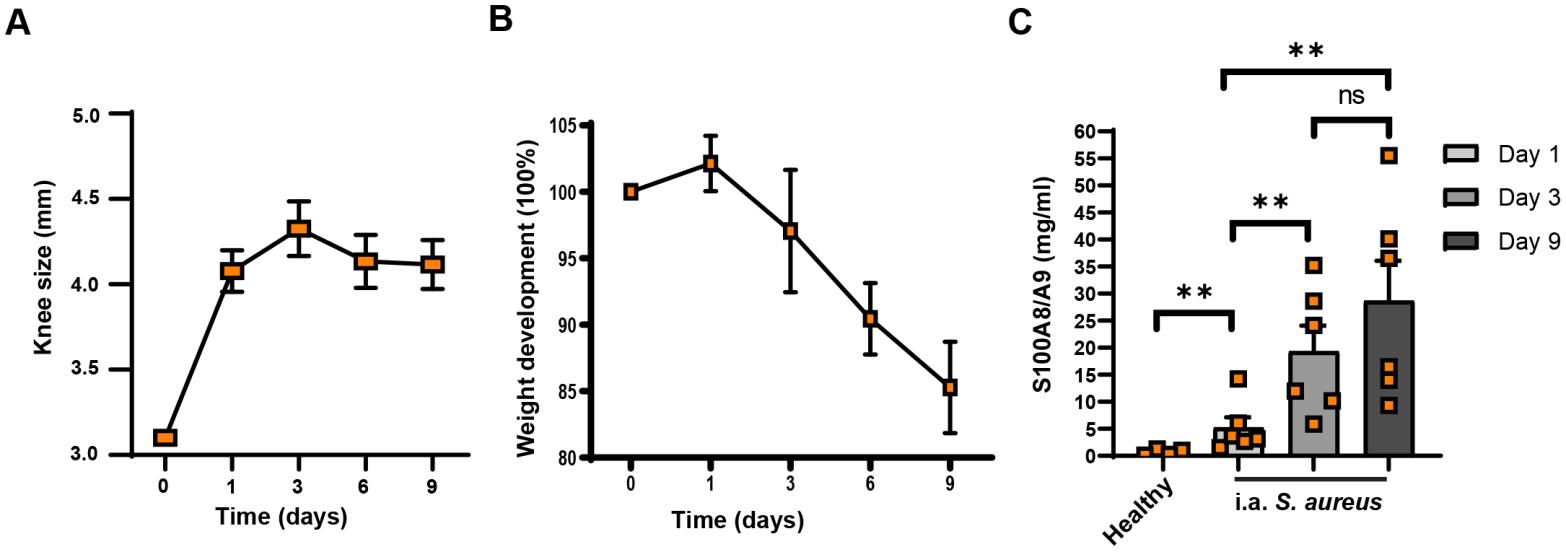
*S100a8/a9* levels correlate with joint swelling in mice. Knee size **(A)**, weight development **(B)**, and *S100a8/a9* plasma levels **(C)** in the NMRI mice upon the intra-articular knee joint infection with *Staphylococcus aureus* (*S. aureus*) Newman wild type (WT). Statistical analyses were performed using the Mann–Whitney U test and the data were represented as the mean ± SEM. **p < 0.01; n.s = not significant.

To understand whether *S100a8/a9* is upregulated in the affected joints in septic arthritis, we first i.a. infected the mice knee joints with *S. aureus* Newman WT strain and measured the *S100a8/a9* protein levels of mice knee homogenates at different time points. After the infection, the knees became inflamed and knee size increased with time (Figure 5A). The weight of infected mice decreased over time (Figure 5B). Importantly, the *S100a8/a9* protein levels steadily increased over time in the *S. aureus* Newman-infected knee joints of mice (Figure 5C) with a median value ranging from 3.415 on day 1 to 18.05 on day 3 and 26.48 on day 9 after infection, as compared to 0.6361 in healthy controls.

### 3.5. Pam2CSK4 upregulated *S100a8/a9* levels in local joints in a monocyte/macrophage-dependent manner

**Figure 6:**
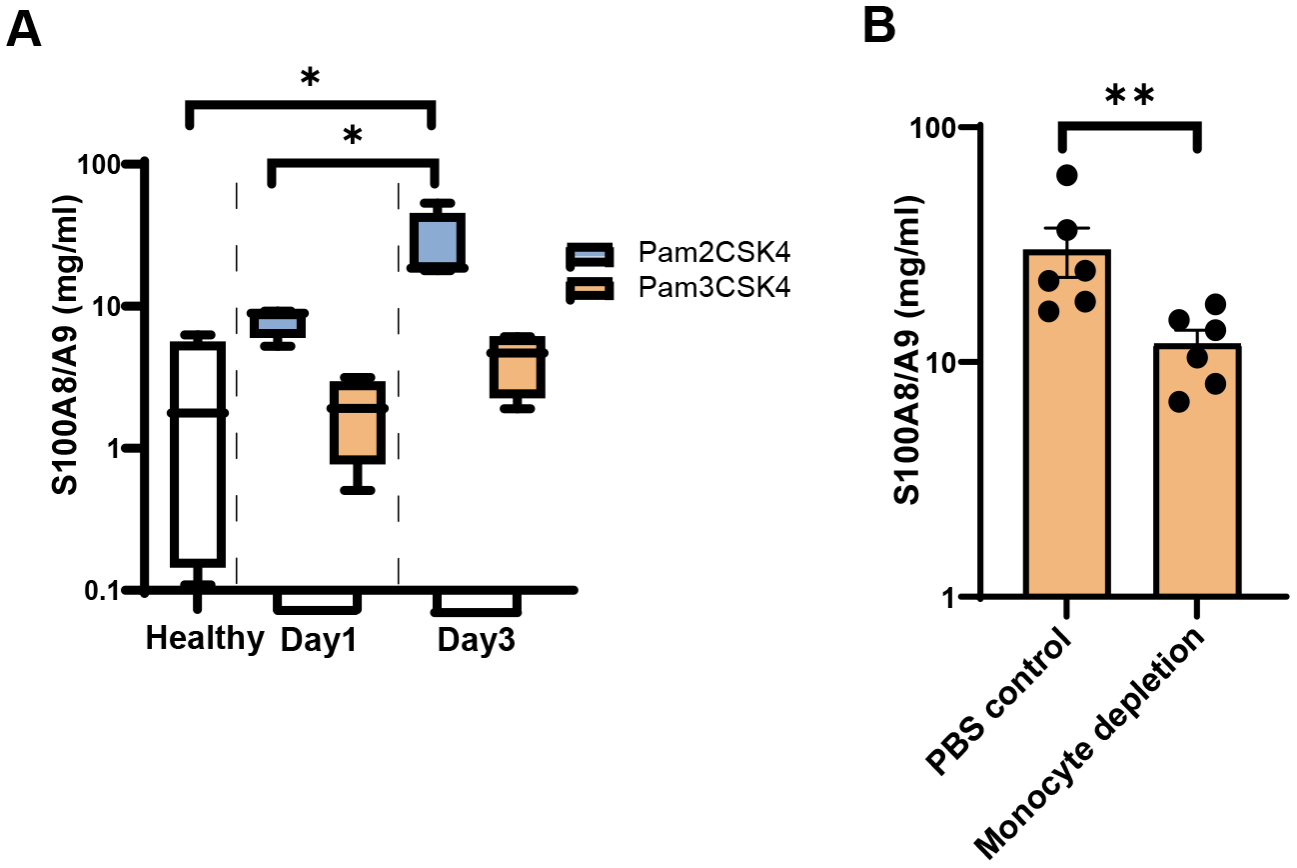
*S100a8/a9* levels in the mice injected with synthetic *Staphylococcus aureus* (*S. aureus*) lipopeptides. **(A)** *S100a8/a9* levels in the knee homogenates of NMRI mice injected with the synthetic lipopeptide Pam2CSK4 or Pam3CSK4. **(B)** *S100a8/a9* levels in the knee homogenates of NMRI mice treated with clodronate liposomes or PBS control liposomes followed by intra-articular injection with the Pam2CSK4 synthetic lipopeptide. Statistical analyses were performed using the Mann–Whitney U test and the data were represented as the mean ± SEM. *p < 0.05; **p < 0.01.

*Staphylococcus aureus* lipoproteins are the most potent bacterial component causing joint inflammation and bone damage in septic arthritis (Mohammad et al., 2019) Here, we i.a injected the mouse knees with synthetic lipopeptides (Pam2CSK4 and Pam3CSK4) to observe whether lipopeptides upregulates *S100a8/a9*. Figure 6A shows a substantial rise in *S100a8/a9* levels in Pam2CSK4 injected knees (knee homogenates) on day 3 compared with the healthy controls. However, no difference was found between samples treated with Pam3CSK and healthy controls (Figure 6A).

*Staphylococcus aureus* lipoproteins-induced joint inflammation is mediated by monocytes/macrophages (Mohammad et al., 2019; Kopparapu et al., 2021). To study whether monocytes/macrophages were responsible for *S100a8/a9* release induced by Pam2CSK4, we depleted monocytes/macrophages through treatment with clodronate liposomes and injected Pam2CSK4 into mouse knees. Significantly lower *S100a8/a9* levels were observed in the knees of monocytes/macrophage-depleted mice compared with the control mice (Figure 6B). Our data suggest that monocytes/macrophages are one of the major immune cells responsible for *S100a8/a9* release in the Pam2CSK4-induced arthritis model.

### 3.6. *S100a8/a9* plasma levels were reduced with antibiotic treatment

**Figure 7:**
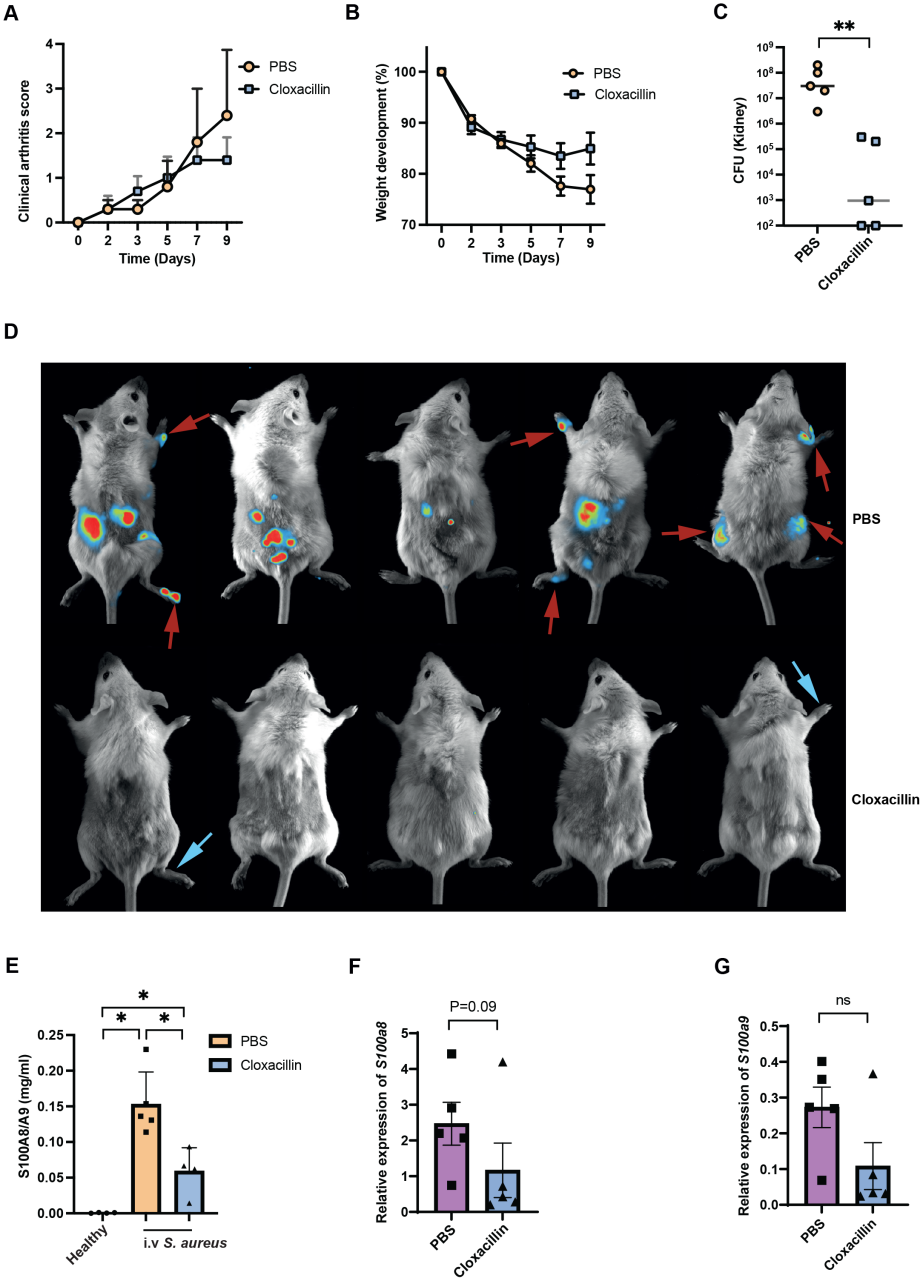
Antibiotic treatment reduces the plasma levels of *S100a8/a9*. Clinical arthritis **(A)**, weight development **(B)**, bacterial counts in kidneys **(C)** and representative images of the *S. aureus* Newman infected NMRI mice treated with PBS (upper panel, red arrow indicates both arthritis and bioluminescent signals) or cloxacillin (lower panel, blue arrow indicates only arthritis) **(D)**. Plasma **(E)**, blood mRNA levels of *S100a8* **(F)** and *S100a9* **(G)** in the *S. aureus* Newman infected NMRI mice treated with PBS or cloxacillin. Statistical analyses were performed using the Mann–Whitney U test and the data were represented as the mean ± SEM. *p < 0.05; **p < 0.01; n.s = not significant.

From the above experiments, we notice the *S100a8/a9* plasma levels are upregulated upon infection. With this, we further investigated if *S100a8/a9* plasma levels can be reduced by the antibiotic treatment. After cloxacillin treatment, the severity of septic arthritis and weight loss percentage stabilized as expected, while they continued to worsen in the control group (Figures 7A,B). Also, bacterial counts in kidneys were significantly reduced in the mice received cloxacillin (Figure 7C). However, there was no significant improvement in the arthritis score on day 9 post-infection. It is known that antibiotics killed *S. aureus* has strong arthritogenic properties and can cause chronic joint inflammation (Ali et al., 2015). To compare the live bacteria load in joints between the cloxacillin treatment group and the PBS control group, we further analyzed the *in vivo* bioluminescent images. We found that on day 9 post-infection, three control mice had both arthritis and bioluminescent signals from corresponding joints. In contrast, two mice from the cloxacillin treatment group had severe joint inflammation but without any bioluminescent signals (Figure 7D). This suggests that cloxacillin treatment was effective in eradicating living *S. aureus* in the joints. However, joint inflammation still persisted, which is considered as a sequelae rather than a clinical manifestation of septic arthritis. Importantly, the infected mice treated with the cloxacillin showed reduced protein expression of *S100a8/a9* compared with the infected mice treated with PBS as shown in Figure 7E. We found that a strong trend toward lower *S100a8* and *a9* gene expression levels in the cloxacillin treatment group (Figures 7F,G).

## 4. Discussion

The hematogenous spread of *S. aureus* to the joint cavity is the most commonly reported route of infection accounting for more than 70% of all septic arthritis cases (Jin et al., 2021). Only 12–17% of patients with *S. aureus* bacteremia developed bone and joint infections (Fowler et al., 1998; Marr et al., 1998). *S. aureus* cell wall debris and cell wall components such as lipoproteins are extremely arthritogenic (Ali et al., 2015; Mohammad et al., 2019, 2022). Therefore, one of the most important treatments for septic arthritis is joint aspiration to flush out the intra-articular pus containing both dead bacteria and infiltrating immune cells (Mathews et al., 2010). It is also known that early treatment of septic arthritis will contribute to a better prognosis of the disease. The early prediction of the high risk of septic arthritis in bacteremia patients will help us to take precautions for those patients and plan the possible interventions together with orthopedicians. In the current study, our data strongly suggest that *S100a8/a9* gene expression might serve as a predictor for septic arthritis. We also show that *S100a8/a9* is highly expressed in the locally infected joints in murine septic arthritis. Staphylococcal lipopeptides increase the *S100a8/a9* levels in local joints and such effect is largely mediated by monocytes/macrophages.

Is high *S100a8/a9* gene expression a causative factor or just a biomarker without any biological meaning for septic arthritis? Calprotectin plays a protective role to combat invading pathogens, as calprotectin is known to chelate the metals such as manganese and zinc that are essential for bacterial survival. Overexpression of calprotectin deprives of the metals in the local infection sites and consequently inhibits *S. aureus* growth (Corbin et al., 2008). By binding to manganese and zinc, S100A8/9 increases *S. aureus* sensitivity to superoxide stress and plays a protective role in a systemic mouse model of *S. aureus* infection (Kehl-Fie et al., 2011). The blood levels of *S100a8/a9* were correlated to the disease severity in sepsis patients (Gao et al., 2015). Additionally, surviving patients had significantly lower *S100a8/a9* levels compared with non-survivors in sepsis (Payen et al., 2008; Gao et al., 2015). Therefore, in our study, the septic arthritis mice may suffer from more severe systemic infection at the pre-arthritis stage compared to those infected mice without septic arthritis and high *S100a8/a9* expression may just reflect a more severe systemic inflammation in those mice. However, it is also known that calprotectin can enhance *S. aureus* virulence by activating the bacterial SaeRS two-component system (TCS) (Cho et al., 2015). TCS controls the expression of over 20 staphylococcal virulence factors including surface proteins and toxins (Liu et al., 2016). Several TCS-regulated surface proteins such as coagulases, fibronectin-binding proteins, and fibrinogen-binding proteins are known to play important roles in the induction of *S. aureus* septic arthritis (Josefsson et al., 2001; Palmqvist et al., 2005; Na et al., 2020). TCS-regulated Staphylococcal leukocidin can damage phagocytes such as macrophages and neutrophils (Münzenmayer et al., 2016). Neutrophils are known to be protective in septic arthritis development (Verdrengh and Tarkowski, 1997). Therefore, it is also possible that high expression of calprotectin activates the *S. aureus* TCS system, which upregulates the expression of surface proteins and toxins in *S. aureus*, consequently leading to more septic arthritis development.

Our data suggest that *S. aureus* is a very strong inducer for *S100a8/a9* expression in both blood and local joints. Indeed, blood levels of *S100a8/a9* increased drastically on day 2 post-infection. Which pathogen-associated molecular patterns (PAMPs) on *S. aureus* are responsible for the upregulation of *S100a8* and *S100a9* in *S. aureus* infection? Bacterial flagellin is known to upregulate *S100a8*/*S100a9* expression in epidermal keratinocytes via TLR5 (Abtin et al., 2010). Staphylococcal lipoproteins were shown to be extremely arthritogenic and joint-destructive in a mouse model (Mohammad et al., 2019). Pam2CSK4 is more potent than Pam3CSK4 in inducing joint inflammation and decreasing bone mineral density (Schultz et al., 2022). Here, our data demonstrated that Pam2CSK4 rather than Pam3CSK4 induced *S100a8*/*S100a9* expression in local joints, and such upregulation was largely mediated through monocytes/macrophages. Nevertheless, slight upregulation of *S100a8*/*S100a9* induced by Pam2CSK4 in local joints hints that there might be some other PAMPs in *S. aureus*, which is much more potent than lipoproteins in upregulating *S100a8*/*S100a9* expression.

Our data clearly show that gene expression levels of *S100a8/S100a9*, rather than protein levels, predict the development of septic arthritis in mice with systemic *S. aureus* infection. This discrepancy may be due to the fact that *S100a8*/*S100a9* are abundantly expressed by neutrophils and stored in specific granules, comprising approximately 40% of cytosolic protein in neutrophils and approximately 40-fold less in monocytes (Edgeworth et al., 1991) Intracellular *S100a8*/*S100a9* are quickly released upon degranulation when neutrophils are stimulated with formyl peptides fLMF from *S. aureus* (Stroncek et al., 2005). Therefore, it is most likely that plasma levels of S100A8/9 reflect the S100A8/9 release from neutrophils rather than gene expression and protein synthesis. Indeed, our correlation analyses also revealed that bone erosion severity by μCT is significantly positively correlated with gene expression levels of both *S100a8/S100a9*, but not with plasma levels of *S100a8*/*S100a9*.

*Staphylococcus aureus* is known to manipulate host immune responses for their survival through disruption of intracellular signaling pathways and transcription factor activation in immune cells (Thammavongsa et al., 2015). At the same time, the interplay between bacteria and host factors determines the induction of infectious diseases such as septic arthritis (Jin et al., 2021). Therefore, distinctive gene expression patterns may exist that distinguish septic arthritis from other invasive *S. aureus* infections. In clinical trials, septic arthritis in patients can be very heterogenous in causative pathogens that might lead to differences in immunological response as well as different stages of infection when patients were enrolled at the emergency unit. Therefore, the mouse model is much better controlled than the heterogenous patient cohort, which means that there is less variation and noise in the later mega-data analyses. Our strategy was to first identify the top candidates in the animal models and thereafter validate them in the patient cohort. To translate the mouse data to the clinical setting, it is crucial to further validate the identified biomarkers in patients. It has been shown that synovial fluid (SF) calprotectin as a biomarker was superior to neutrophil counts in SF and serum CRP levels to discriminate septic arthritis from non-septic inflammatory arthritis diseases such as gout and rheumatoid arthritis (Baillet et al., 2019). Importantly, not only SF calprotectin but also serum calprotectin levels were significantly higher in septic arthritis patients compared to non-septic arthritis patients (Couderc et al., 2019). Calprotectin levels in both SF and blood were also shown to be useful markers to diagnose chronic hip and knee periprosthetic joint infections (Grzelecki et al., 2021). In addition, a very recent report suggests that calprotectin is a promising diagnostic biomarker for the detection of periprosthetic joint infection in a concomitant periprosthetic fracture (Lazic et al., 2022). All this clinical evidence suggests the potential use of S100A8/9 as the diagnostic biomarker for bacterial joint infections. However, it is still unknown whether S100A8/9 can be used as a predictor for septic arthritis in bacteremia patients. Future clinical studies are warranted to further validate our data from animal models.

What is the clinical significance of our findings? It is known that permanent joint dysfunction and disability are associated with delayed presentations and diagnosis (Carpenter et al., 2011). The majority of septic arthritis cases occur through hematogenous spreading (Shirtliff and Mader, 2002; Carpenter et al., 2011; Mohammad et al., 2020). The data from our animal model for septic arthritis suggest that the time window from bacteremia to symptom debut of septic arthritis is about 3–5 days. Identification of high-risk patients for septic arthritis during this time window may significantly contribute to early diagnosis and prompt intervention, which may lead to a significantly better prognosis for patients with septic arthritis.

## Data availability statement

The datasets presented in this study can be found in online repositories. The names of the repository/repositories and accession number(s) can be found at: https://www.ncbi.nlm.nih.gov/, GSE222530.

## Ethics statement

The animal study was reviewed and approved by Jordbruksverket, University of Gothenburg.

## Author contributions

MD performed the experiments, data interpretation, statistics, and wrote the first draft of the manuscript. SS performed the computational analysis of RNA sequencing data, made figures, and wrote the manuscript. ZH, MM, and AJ performed the experiments, reviewed, and edited the manuscript including figures. RP reviewed the data and edited the manuscript. TJ designed and supervised the study, provided funding, and wrote the paper. PKK designed and supervised the study, performed the experiments, data interpretation, made figures, and wrote the manuscript. All authors have read and agreed to the published version of the manuscript.

## Funding

This study was supported by the Swedish Medical Research Council (grant numbers 523-2013-2750 and 2019-01135 to TJ); grants from the Swedish state under the agreement between the Swedish Government and the county councils, the ALF-agreement (grant numbers ALFGBG-823941, ALFGBG-933787, and ALFGBG-965074 to TJ, ALFGBG-770411 to AJ, ALFGBG-926621 to RP), the Wilhelm and Martina Lundgren Foundation to (TJ and 2019-3163, 2022-3950 to PKK); Rune och Ulla Amlövs Stiftelse för Neurologisk och Reumatologisk Forskning to MM, TJ and 2021-283 to PKK; Adlerbertska Forskningsstiftelsen to TJ; E och K. G. Lennanders stipendiestiftelse to MM; Sahlgrenska University Hospital Foundations to MM, AJ, ZH and MD (grant number SU-984324) and PKK (grant number SU-984446); Inger Bendix Stiftelse för Medicinsk Forskning to MM. Magnus Bergvalls Stiftelse (grant number 2022-426 to MM); Petrus och Augusta Hedlunds Stiftelse (grant number M-2023-2079 to MM).

## Conflict of interest

The authors declare that the research was conducted in the absence of any commercial or financial relationships that could be construed as a potential conflict of interest.

## Publisher’s note

All claims expressed in this article are solely those of the authors and do not necessarily represent those of their affiliated organizations, or those of the publisher, the editors and the reviewers. Any product that may be evaluated in this article, or claim that may be made by its manufacturer, is not guaranteed or endorsed by the publisher.
