# Supplementary material for: Gene expression of S100a8/a9 predicts Staphylococcus aureus-induced septic arthritis in mice
Source: Front Microbiol. 2023 Jun 15;14:1146694. doi: 10.3389/fmicb.2023.1146694 (PMC10307981; doi:10.3389/fmicb.2023.1146694)
Supplement: Supplementary file 1 [file Data_Sheet_1.zip › Supplementary File Legends.DOCX]

**Legends to supplementary files**

**Supplementary figure 1. Comparative study of septic arthritis parameters in the NMRI mice infected with Newman wild type or Δ*sortase A/B***

Weight development **(A)**, clinical arthritis **(B)**, survival **(C)**, abscess score **(D)**, bacterial count in kidneys **(E)**, and bone erosion measurement **(F)** of the mice after intravenous injection with *Staphylococcus aureus* (*S. aureus*) Newman wild type (WT) or Newman Δ*sortase A/B*. Statistical analyses were performed using the Mann–Whitney U test and the data were represented as the mean ± SEM. * *p* < 0.05.

**Supplementary file 1.** List of differentially expressed genes from Arthritic (Day 2 & Day 10), non-arthritic (Day 2 & Day 10) *S.* *aureus* infected mice.

**Supplementary file 2.** Functional terms enriched by genes specifically deregulated in Day 2 Arthritic mice from the comparison between Day 2 Arthritis and control mice.

**Supplementary file 3.** Functional terms enriched by genes specifically deregulated in Day 2 non-arthritic mice from the comparison between Day 2 non-arthritic and control mice.

**Supplementary file 4.** Functional terms enriched by genes commonly deregulated in Day 2 arthritic and Day 2 non-arthritic mice from comparisons between Day 2 Arthritis and control mice; and Day 2 non-arthritis and control mice.

**Supplementary file 5.** Functional terms enriched by genes deregulated in Day 2 Arthritic mice from the comparison between Day 2 Arthritis and Day 2 non-arthritic mice.
