# Supplementary figures and images for: Gene expression of S100a8/a9 predicts Staphylococcus aureus-induced septic arthritis in mice
Source: Front Microbiol. 2023 Jun 15;14:1146694. doi: 10.3389/fmicb.2023.1146694 (PMC10307981; doi:10.3389/fmicb.2023.1146694)

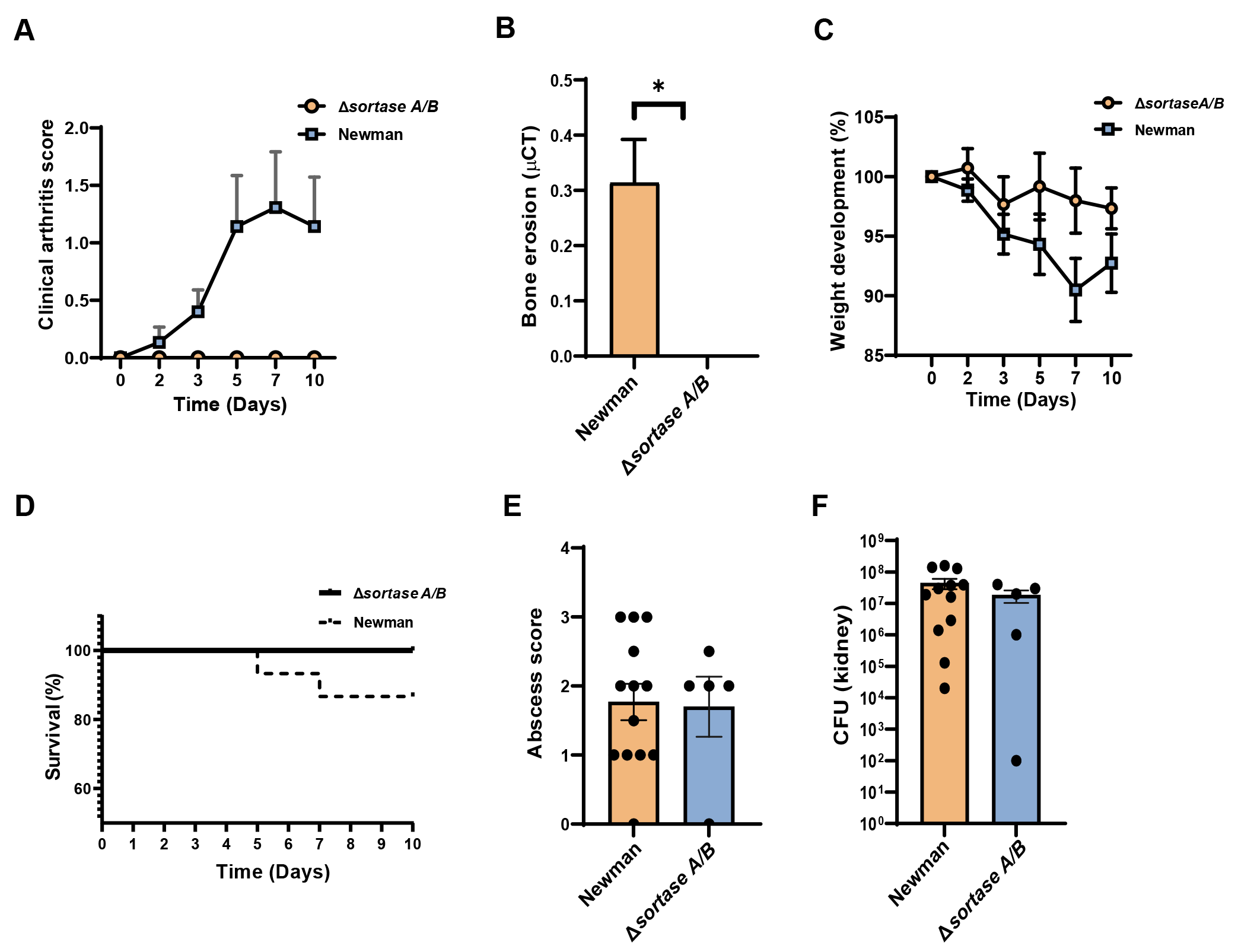

Supplement: Supplementary file 1 [file Data_Sheet_1.zip › Image 1.TIF]

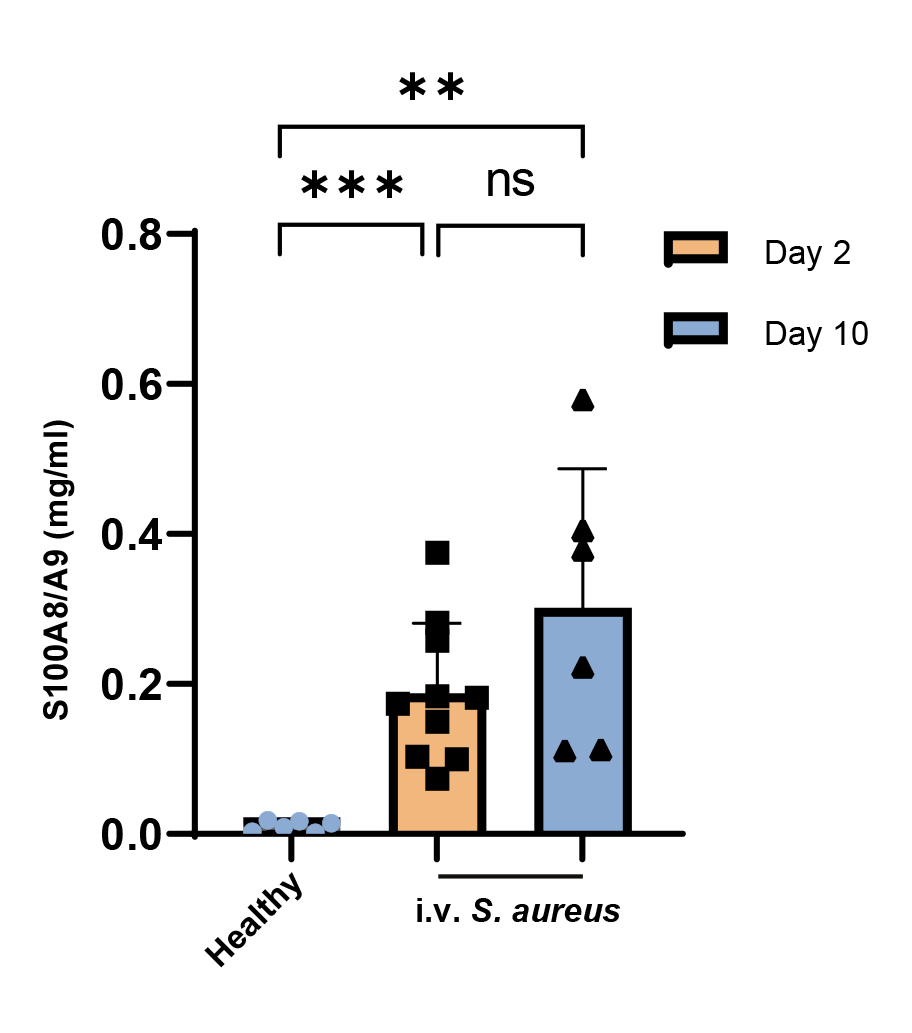

Supplement: Supplementary file 1 [file Data_Sheet_1.zip › Image 2.TIF]

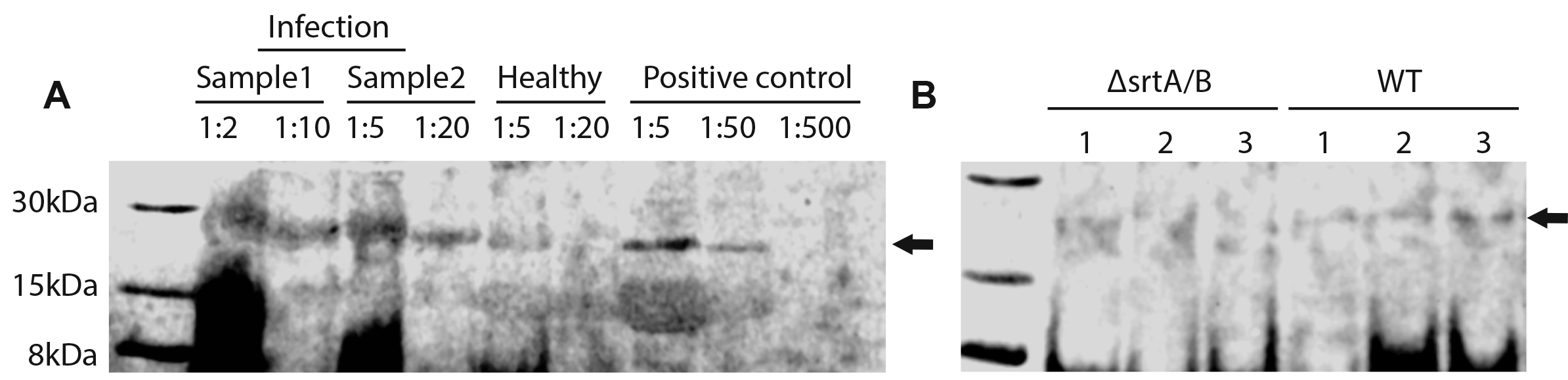

Supplement: Supplementary file 1 [file Data_Sheet_1.zip › Image 3.TIF]
